# Why do patients refuse trichiasis surgery? Lessons and an education initiative from Mtwara Region, Tanzania

**DOI:** 10.1371/journal.pntd.0006464

**Published:** 2018-06-14

**Authors:** Katherine M. Gupta, Jennifer C. Harding, Majid S. Othman, Shannath L. Merbs, Emily W. Gower

**Affiliations:** 1 Children’s Hospital of Philadelphia, Philadelphia, Pennsylvania, United States of America; 2 Helen Keller International, Dar es Salaam, Tanzania; 3 Kongwa Trachoma Project, Kongwa, Tanzania; 4 Wilmer Eye Institute, Johns Hopkins School of Medicine, Baltimore, Maryland, United States of America; 5 Gillings School of Global Public Health, University of North Carolina at Chapel Hill, Chapel Hill, North Carolina, United States of America; University of Tennessee, UNITED STATES

## Abstract

**Background:**

Trachomatous trichiasis is one of the leading causes of preventable blindness worldwide. A relatively simple surgery can spare vision. Although this surgery is usually performed free of charge in endemic regions, multiple studies indicate that surgical refusal is common. Prior studies have attempted to examine these reasons, although they generally rely on patient recall months to years after the surgery was offered. This study set out to determine major decision-making factors at the time of refusal. In addition, this study looked for ways to help increase surgical uptake by targeting modifiable factors.

**Methodology/Principal findings:**

We used a combination of focus groups, interviews with community health workers, and individual interviews with trichiasis patients who refused surgery to understand their decision-making.

We found that several factors influenced surgical refusals, including misconception regarding recovery time, inability to find a post-surgical caregiver, and the time of year of the surgical campaign. Fear of the surgery itself played a minimal role in refusals.

**Conclusions/Significance:**

Trichiasis patients refuse surgery for many reasons, but a large percentage is due to lack of information and education, and is, therefore, modifiable within the structure of a surgical outreach project. To address this, we developed a “frequently asked questions” (FAQ) document aimed at community health workers, which may have helped to decrease some of the misconceptions that had led to prior refusals.

## Author summary

Many international health interventions in resource-limited settings involve treatments that are fully funded by donor organizations and, therefore, available for free to those in need. Trachoma, a bacterial eye disease that can cause blindness if left untreated, has frequently been the target of surgical outreach programs. However, even when the surgery is offered for free, a substantial number of eligible patients refuse surgery. This study explored why eligible patients did not present for surgery. When findings indicated that many patients did not understand the intervention and feared that it would be a major procedure or involve a long recovery time, we created a frequently asked questions document to address these misconceptions directly. This document was used at the surgical recruitment stage to better educate patients and community health workers, and it likely helped increase turnout for surgery. This study has implications beyond just eye surgery, and suggests steps that other global health interventions may want to consider to identify and address barriers uptake.

## Introduction

Trachoma is an eye disease caused by the bacterium *Chlamydia trachomatis*. It is common in areas of the world that lack access to health care and clean water. Repeated infections cause recurrent conjunctivitis, which then leads to significant scarring of the eyelid. This scarring causes contraction of the inner surface of the eyelid, pulling the eyelashes inward to rub against the eye in a process known as “trichiasis”. The resulting irritation and damage to the cornea makes trachomatis trichiasis one of the leading causes of preventable blindness in the world [1]. According to the World Health Organization (WHO), nearly 2 million people worldwide have vision loss due to trichiasis, with a further 190 million at risk and living within trachoma-endemic regions[2]. As part of their “SAFE” strategy for the elimination of blinding trachoma, WHO recommends surgery for the correction of trichiasis; this procedure has the ability to relieve pain from corneal trauma and prevent further vision loss. In addition, recent data from Ethiopia suggest that trichiasis surgery improves quality of life irrespective of post-surgery visual acuity improvement[3]. This surgery can be performed in an outpatient setting in less than half an hour, and is often performed free of charge to patients in areas with high need, with costs typically underwritten by donors. Despite the availability of free services, anecdotal and published evidence indicate that surgical refusal rates remain high [4,5]. This suggests that important drivers besides cost may affect the decision-making of trichiasis patients. Better understanding of these drivers can help tailor future interventions and large-scale trachoma elimination programs, making them more effective for both patients and donors alike.

The goal of this project was to identify why people with trichiasis refuse free, corrective surgery. Reasons for refusal were obtained using interviews with community health workers (CHWs) (many of whom had close relationships with their villagers and were knowledgeable regarding their reasons for not showing up) and with the individuals who refused surgery. Although literature exists on patient satisfaction years after this procedure,[4,6–8] there is a lack of information obtained from patients at the time of their decision whether to undergo surgery. By conducting interviews on the same day that patients refused surgery, this study has the ability to examine patient reasoning with less recall bias.

## Methods

This refusals study utilized the patient population recruited for the Partnership for the Rapid Elimination of Trachoma (PRET) Surgical Trial.

### Ethics statement

The PRET Surgical Trial parent study was approved by the Johns Hopkins Medical Institutions and Wake Forest School of Medicine Institutional Review Boards and the National Institute for Medical Research in Tanzania. Participants who were interviewed regarding their reasons for surgical refusal (which constituted minimal harm and had no “intervention” group) provided implied oral consent in their native language (Kiswahili or Kimakonde) at the time when they were asked if they would like to talk with the interview team.

### PRET Surgical Trial

The PRET Surgical Trial was a randomized controlled trial designed to investigate the performance of a novel surgical instrument, the TT clamp,[9] developed specifically for use in trichiasis surgery. It compared one of the WHO-endorsed procedures, the bilamellar tarsal rotation procedure (BLTR), [10] performed using the standard method (two hemostats) to BLTR using the TT clamp. The study was conducted in Mtwara Region, southern Tanzania, which is a trachoma-endemic area. Surgeries took place between June and October 2009. Results from the primary study were previously reported[11].

The PRET trial identified study participants at the village level through a multistep process. Trained district-level health personnel, accompanied by study team members, visited each study village on two separate occasions before the surgical outreach, once for an informational session and then again for a screening day. During the informational session, both village leaders and CHWs were familiarized regarding the process of TT treatment and were mobilized to assist on the upcoming screening and surgery days. In Mtwara region, CHWs are lay people without formal health education who act as point people for many different health initiatives and interventions. Village leaders and CHWs were tasked with spreading the information among their constituents. On the subsequent screening day, all residents with any eye problem were invited to receive an evaluation by a trained nurse, who determined a diagnosis and recorded patient demographics in a screening log.

Villagers who were identified as having trichiasis at the screening were advised to return on a specific date for free, corrective surgery. Surgeries were performed by surgeons with experience performing trichiasis surgery in this region at a central health dispensary, which generally served several villages within a few-mile radius. Upon arrival at the surgery site, all eligible trichiasis-surgery patients were invited to participate in the PRET Surgical Trial. If they were interested, they were consented and then randomized to receive surgery using either the standard BLTR procedure or BLTR with the new instrument. Patients undergoing surgery who either were ineligible or declined consent to participate in the trial received surgery using the standard BLTR method. The names of all patients receiving surgery were recorded in a surgical log, which was maintained separately from the screening log for each village.

### Refusals study

The refusals ancillary study ran concurrently with the PRET Surgical Trial from 7 July through 18 August 2009, which encompassed two focus groups held in villages in Masasi district and 19 surgical days in Mtwara Rural district.

We first conducted two focus groups with trichiasis patients in Masasi district, in villages where surgeries had been performed two weeks prior. The goal of these discussions was to determine major themes of surgical refusal to help guide day-of-surgery individual interviews in Mtwara Rural district.

Subsequently, during trichiasis surgical camps in Mtwara Rural district, we identified subjects we considered “refusals” because they screened positive for trichiasis but did not present for surgery. For the time period of this study, 575 individuals were identified as having trichiasis through village screening days, of which 464 underwent corrective surgery. Out of the 111 subjects who did not receive surgery, five had presented as instructed to the health dispensary, but PRET staff determined they had been inappropriately screened and did not have trichiasis, leaving a total of 106 refusals (18.4%). The count of refusals was determined by comparing the screening log to the surgical log for that village.

We determined the names of refusals from cross-referencing the logbooks and then met with the associated CHWs to learn information regarding any known reasons for the absences. Using a convenience sample, we then conducted in-person interviews with patients who lived within a short walking distance of the health dispensary. We conducted interviews using an open-ended question format; patients initially were asked simply why they had not presented for surgery. Subsequently, their answers were explored with particular emphasis on themes that had emerged during the Masasi focus groups (such as anecdotal experiences of friends and family with trachoma surgery, and their understanding of why a surgery was necessary and how it differed from other ways to manage trichiasis). A PRET study team member fluent in Kiswahili and English, and a community health worker who could provide translation between Kiswahili and Kimakonde (the local language) when needed, conducted the interview discussions, translating the findings into English for the visiting research team member, who then guided further questioning. Interviewers were instructed to maintain a non-judgmental style and were told that the goal of the interviews was to determine reasons for refusal, not to attempt to convince patients to consent to surgery. However, for ethical reasons, during the course of the interview if the participant expressed interest in undergoing the procedure, he/she was offered surgery. Patients who initially refused surgery were still counted as refusals for this ancillary study, even if they decided to undergo surgery after their interview.

Using the results from focus groups, CHWs, and individual interviews, we prepared a frequently asked questions (FAQ) sheet and distributed it to CHWs at informational meetings between district teams and village leadership, with the goal of enabling village leaders to better educate their community regarding trichiasis surgery before the surgical outreach began.

## Results

We conducted two focus groups in Masasi district: nine people who had refused trichiasis surgery when it was offered in their village and six people who initially refused surgery, but then changed their minds and received the procedure on a subsequent date. In Mtwara Rural district, we interviewed CHWs to obtain information on 89 refusals (84% of all refusals during the study time period) and conducted in-person interviews with 13 individuals who refused surgery.

### Masasi district retrospective refusal focus groups

Among the patients interviewed in Masasi district who had not received surgery, almost half (four of nine) reported confusion regarding the date that the surgical team was to be present. This sub-group expressed interest in having the procedure at a later date. For the rest, several themes emerged. The most common reason given for not undergoing surgery was anecdotal poor experiences from acquaintances and family, some of whom had previously undergone trichiasis surgery, and some of whom had clearly undergone other types of eye surgery (e.g. cataract surgery). There was a widespread belief that the surgery itself would cause more problems. Others believed that because the pain associated with their trichiasis could be managed non-surgically (with medications and epilation), surgery was not necessary and would offer no additional benefits. Finally, several people who declined surgery discussed fear of the pain associated with surgery as a major deterring factor.

In the focus group of people who had initially refused, but then later requested and received surgery, fear and skepticism were the predominant reasons mentioned for initial refusal. Many admitted that they wanted to see how others in the village did after surgery, and when they saw the rapid recovery of their neighbors, they wanted the surgery for themselves. Members of this group thought that better education surrounding the surgery would lead to higher participation rates. Interestingly, several people in this group said that after their surgery they have encouraged others to get the surgery, citing its benefits and short recovery time. This was in contrast to reports from the group that continued to refuse surgery, where several members insisted that they had heard negative things about the procedure and recovery process from villagers who had undergone surgery.

### Mtwara day-of-surgery refusals

For those patients in Mtwara Rural who did not present for surgery, information obtained through in-person interviews and conversations with CHWs revealed several trends. For in-person interviews, the most common reason given for refusing surgery was lack of a post-operative caregiver. This rationale was augmented by the widespread belief that the recovery period from surgery was up to six months, during which time the patient would be unable to cook, help with household chores, or farm. However, even those who understood that the period of incapacitation was very short encountered difficulties, with numerous women reporting that they were unable to find anyone to cook for them for even one night. These findings were roughly mirrored in the responses given by CHWs, although workers were more likely to state that it was the perceived long recovery period (rather than a lack of caregiver) that prompted the refusal.

PRET surgery recruitment in Mtwara Rural district coincided with cassava harvest. Many of the refusals who were unable to be interviewed in person were out working on their farms. Although this precluded the patients themselves from being interviewed, CHWs reported this season to be a significant factor in patient refusals.

A less common, but not infrequent reason for refusal was lack of understanding regarding the need for trachoma surgery if medication was already giving effective pain relief. Many people with trichiasis either apply topical antibiotics or epilate their eyelashes to provide relief from the pain of their condition. The problem with both of these methods is that while they temporarily relieve the pain, they do not address the underlying problem (eyelashes rubbing against the surface of the eye) and may not prevent blindness [12]. In the absence of accurate information about the etiology of their disorder and how surgery could correct the problem, many villagers were choosing medication over surgery.

The procedure to correct trichiasis was described as *upasuaji mdogo* (literal translation: small surgery), which led many people to associate it with other types of surgery with which they had personal or family experiences. A number of anecdotes that subjects recounted in their decision to refuse surgery clearly involved other procedures, most often relating to childbirth or abdominal complaints. One person interviewed clearly stated she had been confused by the word “upasuaji” until it was further explained.

No one interviewed individually gave fear of pain or fear of the surgery itself as a reason for refusal. In contrast, CHWs attributed some refusals to fear, although it was still not a leading reason for refusal. For the purpose of further exploring the issues surrounding fear of surgical pain, non-refusal patients were informally interviewed while waiting their turn for surgery. They reported that their trichiasis was so painful that they didn’t mind temporary surgical pain if it would bring them more permanent relief. Fear of the surgery among this group tended to manifest itself as fear of the more long-term consequences, such as the inability to see afterwards or inability to care for their family for many weeks or months.

In two cases, villagers refused the surgery because they were already blind and no longer experienced pain from the in-turned lashes. These subjects felt that, because the surgery would not substantially restore the vision they had already lost, they would not benefit from the procedure.

Summaries of reasons for surgical refusal are given in Tables 1 and 2. A comparison of findings in focus groups and in individual interviews is given in Table 3.

**Table 1:** Day-of-Surgery reasons for refusal reported by community health workers.

Reason	Number^a^ (% out of 89)
Long recovery time	35 (39)
At the farm	21 (24)
Lack of caregiver	14 (16)
Fear	8 (9)
Illness (patient or family member)	3 (3)
Travelling away from the village	3 (3)
Didn’t want repeat surgery	2 (2)
Doesn’t live in screened village	1 (1)
Prefers to use medication	1 (1)
Unknown	19 (21)

^a^ Numbers do not add up to 100% because some patients gave multiple reasons for refusal

**Table 2:** Day-of-Surgery reasons for refusal given by trichiasis patients.

Reason	Number^a^ (% out of 13)
Lack of caregiver	6 (46)
Long recovery time	4 (31)
Not experiencing pain from trichiasis	4 (31)
Didn’t know the date of surgery	3 (23)
Already blind	2 (15)
Misunderstanding regarding the word “surgery”	1 (8)

^a^ Numbers do not add up to 100% because some patients gave multiple reasons for refusal

**Table 3:** Reasons for refusal: Comparison between focus groups and Day-of-Surgery interviews.

Focus Group Reasons For Refusal	Day-of-Surgery Reasons For Refusal
• Poor anecdotal experiences with surgery • Belief that surgery was not necessary if pain relief was achieved with non-surgical methods • Fear of the pain of surgery	• Lack of post-surgical caregiver • Beliefs regarding long recovery time • Need to help on the farm for harvest season • Belief that surgery is not necessary if pain relief is achieved with non-surgical methods • Misunderstanding of the word “surgery” to mean a more major procedure

### Patient education tool

When it became apparent that one of the primary drivers of high refusal rates was lack of understanding around trachoma, trichiasis, and the surgery, we created a frequently asked questions sheet (FAQ, see S1 and S2 Appendix) and distributed it to CHWs at the informational meetings between the district team and the village leadership. We designated the CHWs to serve as the primary educators of potential surgical candidates because we felt the candidates would have more trust in members of their own village than in district and study team members, who were not local to the community. We felt that if the CHWs were properly educated about the procedure and why it was necessary, they could share their knowledge to potential patients and to anyone else who had questions or concerns. The FAQ addressed issues such as how trichiasis leads to blindness, how the surgery is more effective than medication, the length of the recovery time, and the fact that the surgery does not involve the eyeball, only the eyelid. The FAQ was distributed for the remainder of the PRET recruitment period following the end of the refusals study. Reports from the field indicated that it was successful in reducing the number of refusals and increasing health worker knowledge about the procedure, although no formal comparison of refusal rates was performed.

## Discussion

This study found that important misinformation exists that limits people from deciding to undergo trichiasis surgery. These findings are applicable not only to trichiasis surgery programs, but to a broad range of health-related activities implemented at the community level, particularly in developing country settings where access to medical information is limited. In-person interviews with trichiasis patients and discussions with CHWs provided important insights into how inaccurate information and societal pressures can impact the effectiveness of a well-intentioned health care initiative. One key addressable concern was the pervasive belief that trichiasis surgery required a prolonged recovery time, which was a significant deterrent to undergoing surgery. Many believed that their recovery would take months, and that during that time they could not work. In a society that survives on subsistence farming, a months-long recovery period could have substantial economic consequences. This concern was especially apparent during the timing of this study, which coincided with cassava harvest season. In actuality, the recovery time is remarkably short, with patients cleared to remove their bandages the morning after surgery, and able to farm within a few days, even while stitches are still in place.

Misconceptions about the recovery period highlighted a general paucity of experience with the formal health care system as well as a specific lack of information regarding this particular surgery. Through interviewing the CHWs (who met with district team personnel prior to the screening days), it became clear that they wanted to convince their neighbors to have surgery, but were not armed with enough knowledge to counteract misconceptions. It was this finding that prompted the idea of an educational tool that could be implemented for future surgical screenings in an effort to reduce the rate of refusals.

Roughly 75% of PRET trial participants were middle-aged or elderly women; these women are traditionally charged with most of the household tasks and are unable to spend time being incapacitated after surgery. Even among people who understood that the recovery period is short, a substantial proportion indicated that even a few days being unable to work and provide for their families was not feasible, and some indicated that they had no one to help them for even one night. 70–80% of trichiasis patients require bilateral surgery and, hence, would be unable to see for the night of surgery due to the bandaging. For women living alone, this can make surgery unattainable. Unfortunately, these single women are among the most in need, since they likely would have fewer options for care if they become blind from trichiasis. While these rural communities are closely-knit, this finding highlights the fact that stronger support systems are needed to ensure surgical services for those most in need. One method is to provide a structure wherein surgical services are offered again two weeks later, such that the patient can have one eyelid corrected at a time. Research into other methods that would provide better support structures for these most at risk individuals is needed.

An interesting contrast between the focus group interviews in Masasi district and the individual interviews in Mtwara Rural district was the number of anecdotes highlighting poor health outcomes of family members among villagers in Masasi. In this area, trichiasis surgery has been provided for some time, and many people in the focus groups mentioned the bad experiences of friends or family as a reason why they were refusing surgery (e.g., my uncle had this surgery and went blind; my mother had this done and she got worse). In Mtwara Rural, an area that was generally surgery-naïve at the time of this study, no personal eye surgery anecdotes were given as reasons for refusal. It seems that in areas where no one has had the surgery before, there is little opportunity for the potential bad experiences of others to play a powerful role in limiting future surgical uptake. This study highlights the importance of good initial surgical outcomes in programmatic settings, as they are likely to set the standard for future surgical participation in the community. Hence, we strongly urge programs to take all possible steps to provide the highest quality surgery possible.

This study is unique not only in the fact that it interviewed refusals at the time of surgery but also because it surveyed a population for which data also exist regarding satisfaction with trichiasis surgery and reported barriers to surgical uptake. At the follow-up visit two years after surgery, members of the PRET study staff interviewed nearly 500 participants in the Mtwara Rural district regarding their perceptions of the surgery and their outcomes. They found that 86% of patients were “very satisfied” overall, and that 83% felt that surgery had improved their quality of life. In addition, 97% said that they would recommend this surgery to others [6]. This is an optimistic finding, given the weight that anecdotal experience had during our focus groups in Masasi Rural district. There is also concern that as these regions become more familiar with this type of surgery, there is more opportunity for surgical recipients to dissuade their family and neighbors based on poor prior experience. Moving forward, it may be informative to assess current programs in this region to see how improved perceptions about surgery have changed surgical uptake over time.

At the two-year visits, PRET surgical trial participants also were asked to recall if anything made it difficult to obtain the surgery. Only 11% of those surveyed reported facing challenges in obtaining the surgery (although significantly more women reported difficulties than men), and the most common reasons reported were farming, cooking, and not being able to find a suitable caregiver [6]. These reasons, elicited two years post-surgery, are consistent with our findings on the day surgery is offered and serve to reinforce that these are some of the main barriers to overcome to increase rates of surgical uptake.

This study has some important limitations. Due to the design of the study and the logistics of interviewing refusals, in-person interviews were only able to be obtained with those living within a short distance of the surgical site. The effect of this bias cannot be measured, but is presumed to underestimate the effect that travel distance might have played on a patient’s decision not to undergo surgery. A previous study noted that lower surgical refusal rates correspond to shorter distances between patients’ homes and the site of surgery[13], which suggests that some of our patient refusals may have attributed their decision not to undergo surgery to long travel distances. This study also looks at a single region in rural Tanzania, and so conclusions about family/caregiver relationships, the role of women in household tasks, and concerns related to specific crop harvests cannot necessarily be generalized to other settings.

However, subpar utilization of available health services is not an issue specific to Tanzania or to trichiasis surgery programs. In reality, it is encountered worldwide, and may be a major impediment to improving health outcomes in areas with low health care literacy and unfamiliarity with health care infrastructure. Lack of patient education and mistrust of the health care system has been implicated in refusals for interventions as diverse as lumbar punctures[14], hospital births and cesarean sections [15–17], cataract surgery [18], malaria prophylaxis [19,20], and general surgical procedures [21]. This study suggests that the educational deficits surrounding these interventions can be lessened if programs and providers undertake enhanced training of CHWs and village leaders. Based on our findings, we expect that addressing these knowledge gaps has the potential to make large-scale interventions more effective.

## Supporting information

S1 Appendix Frequently asked questions (FAQ) document (English). (DOCX)

S2 Appendix Frequently asked questions (FAQ) document (Kiswahili). (DOCX)
