# Supplementary material for: Why do patients refuse trichiasis surgery? Lessons and an education initiative from Mtwara Region, Tanzania
Source: PLoS Negl Trop Dis. 2018 Jun 14;12(6):e0006464. doi: 10.1371/journal.pntd.0006464 (PMC6001945; doi:10.1371/journal.pntd.0006464)
Supplement: S1 Appendix — (DOCX) [file pntd.0006464.s001.docx]

**S1 Appendix**

**FOR VILLAGE HEALTH WORKERS**

**FREQUENTLY ASKED QUESTIONS AND ANSWERS ABOUT TRICHIASIS SURGERY**

**TO SHARE WITH PATIENTS**

**What is trachoma?**

Trachoma is an eye disease that is very common in Tanzania, especially in places that don’t have access to clean water. It is caused by bacteria.

**What is trichiasis?**

After repeated trachoma infections, the eyelid changes shape and the eyelashes turn inward and rub against the eyeball. Trichiasis is when the eyelashes turn inward and rub on the eye.

**Why are we doing these surgeries?**

Trichiasis can cause blindness if it is not fixed. Also, having surgery will help to stop the pain caused by lashes rubbing against the eye..

**When will the doctors come to do the surgery?**

The doctors will be coming to your area for one day in the near future. If you do not have surgery on that day it will be many months before surgery is available again. Ask your village health worker when the doctors will come to your village. Your village health worker will remind you a couple of days before the surgery day.

**How does this surgery fix the trichiasis?**

The surgeon makes a small cut across the upper eyelid and then stitches the cut back together. This pulls the eyelashes so that they face outwards, instead of inwards towards the eye.

**Does the surgery involve the eye itself?**

No. This surgery only involves the eyelid. The eye itself will not be cut or removed.

**How long does the surgery take?**

Each eye takes about 20 minutes to fix, but you may need to wait your turn to have the surgery. It is possible that you will be at the health center or dispensary for many hours.

**What will happen right after the surgery?**

After the doctor has finished, they will bandage your fixed eyes. If both eyes were operated on, both eyes will be bandaged. You will not be able to see through the bandages. You will be given medication afterwards to use at home. You will be helped home by a family member or your village health worker. On the night of the surgery, you will need help at home to cook and wash.

**When will I be able to see again after surgery?**

You will be able to see as soon as the bandages are removed. The bandages are removed on the morning after surgery. The only reason that you cannot see right after surgery is because of the bandages; your eyes still work.

**When will I be able to return to work after the surgery?**

After the bandages are removed, you can return to work whenever you want. Most people rest for one or two days. You can cook for your family on the day after surgery. You should be able to go to the farm after a few days.

**How long will I need to be nursed after the surgery?**

You will need help on the night of the surgery when your eyes are bandaged. You will need someone to help you get home and to cook for you. After your bandages are removed the next morning, you won’t need any more nursing.

**What should I do if I live alone?**

You will only need help on the night of the surgery. You should find a friend or family member who can help you for that night. The next morning, after your bandages are removed, your nurse can go back home.

**When do I need to see the doctor after surgery?**

You need to return after 2 weeks to have the stitches removed. You can go back to work even while the stitches are in place. If you are concerned about anything after surgery, you can visit your health clinic or dispensary at any time.

**Why is having the surgery better than just taking medicine or pulling out my eyelashes?**

Taking medicine stops the pain, but doesn’t prevent the eyelashes from damaging the eye, and so it doesn’t prevent blindness. Pulling out your eyelashes will only fix the problem for a short time. When the eyelashes grow back, they can cause more pain. This surgery will fix the trichiasis so that you don’t have pain anymore and if you still are able to see it will help to make sure your vision does not get worse from trichiasis.

**Will this surgery allow blind people to see again?**

No. This surgery will not fix blindness, but it can prevent you from losing more sight if you are not totally blind. Some blind people choose to have the surgery anyway to stop the pain and tears.

**Will the surgery hurt?**

At the beginning of the surgery, you will get an injection of pain medication. This injection will hurt for a short time. After the injection, you won’t feel pain for the rest of the surgery. Your eyelids may be swollen or hurt for a few days after the surgery. If you want, you can take a pain medication like Panadol.

**Does the surgery cost anything?**

No. This surgery and the medication afterwards are free.

**Will this surgery fix all eye problems?**

There are many types of eye problems. This surgery will only fix trichiasis. If you have cataracts or glaucoma, you will need to go to a hospital to see a different doctor. If you have trichiasis and another eye problem, we will only be able to fix the trichiasis.
