# Supplementary material for: Why do patients refuse trichiasis surgery? Lessons and an education initiative from Mtwara Region, Tanzania
Source: PLoS Negl Trop Dis. 2018 Jun 14;12(6):e0006464. doi: 10.1371/journal.pntd.0006464 (PMC6001945; doi:10.1371/journal.pntd.0006464)
Supplement: S2 Appendix — (DOCX) [file pntd.0006464.s002.docx]

**S2 Appendix**

***KWA WAJA (WAHUDUMU WA AFYA KIJIJINI)***

**MASWALI NA MAJIBU KUHUSU UPASUAJI MDOGO WA VIKOPE**

**KWA KUSAIDIA KUELIMISHA WAGONJWA WA KIJIJINI KWAKO**

**Trakoma ni nini?**

Trakoma ni ugonjwa unaosababishwa na vijidudu. Sio ugonjwa wa ajabu, ni wa kawaida sana nchini Tanzania na unapatikana hasa kwenye maeneo ambayo uwezekano wa kupata maji safi na salama ni mgumu.

**Ugonjwa wa Vikope ni nini?**

Ugonjwa wa trakoma unapojirudiarudia unasababisha kifuniko cha jicho kubadili umbo lake na kupelekea vikope kujikunja kwa ndani na kusugua gololi ya jicho. Kwa hiyo vikope vinapojikunja kwa ndani na kusugua gololi ya jicho ndivyo hivyo ugonjwa wa vikope unavyopatikana.

**Kwa nini tunafanya upasuaji mdogo?**

Vikope kama havitarekebishwa vinasababisha upofu. Ili kuepuka upofu na maumivu ambayo mtu anayapata kutokana na vikope kusugua gololi ya jicho ndio maana tunafanya upasuaji mdogo wa kurekebisha kope.

**Madaktari watakuja lini kufanya upasuaji mdogo?**

Madaktari watakuja siku za karibuni na huwa ni siku moja. Inabidi kufanya kila liwezekanalo mgonjwa afanyiwe upasuaji katika siku hiyo moja ambayo madaktari watakuja. Mgonjwa akikosa kufanyiwa upasuaji siku hiyo basi itabidi asubiri kwa muda mrefu. Mgonjwa anapaswa kumuuliza WAJA au muhudumu wa afya kujua upasuaji unafanyika lini, pia WAJA watamkumbusha mgonjwa kabla ya siku ya upasuaji kufika.

**Ni jinsi gani upasuaji mdogo unarekebisha vikope?**

Madaktari ambao ni wataalam wa upasuaji, wanachana kidogo sehemu ya kifuniko cha juu cha jicho, wanarekebisha kifuniko hicho na kurudia katika hali yake ya kawaida. Baada ya kufanya hivyo kifuniko cha juu cha jicho hakitasababisha tena kope kujikunja ndani ya jicho. Upasuaji huu hauna maumivu makali kwa sababu madaktari wanatumia ganzi kuzuia maumivu.

**Upasuaji huu unahusisha jicho lote?**

HAPANA, upasuaji huu hauhusishi jicho lote. Jicho haliguswi, wala halitolewi. Kinachoshughulikiwa ni KIFUNIKO cha jicho tu.

**Upasuaji mdogo wa vikope unachukua muda gani?**

Upasuaji huwa unachukua mpaka dakika ishirini (20) kwa kila jicho. Ila utasubiri kwa muda mrefu kidogo kutokana na shughuli za usajili ili kupata namba ya daktari atakayekufanyia upasuaji. Vile vile, utachukua muda kusubiri foleni. Hivyo unatakiwa kujipanga kuwepo kwa siku nzima zahanati.

**Kutatokea nini baada tu ya upasuaji mdogo?**

Baada ya daktari kufanya upasuaji mdogo, watakufunga bendeji kwenye jicho au macho yote (kama yote yalirekebishwa). Ni kweli hutaweza kuona baada ya kufungwa bendeji kwa sababu bendeji itakuwa imekuzuia. Baada ya upasuaji kwa kawaida huwa unapewa dawa za kutumia, pia wahudumu wa afya watakusaidia kurudi nyumbani na familia yako pia itakusaidia kwa siku ile ambayo bendeji inakuzuia kufanya kazi ndogondogo.

**Ni kipindi gani kitapita baada ya upasuaji ili niweze kuona tena?**

Mara tu bendeji itakapotolewa utaweza kuona tena. Bendeji utatoa mwenyewe nyumbani, kesho yake asubuhi baada ya upasuaji. Baada ya upasuaji macho huwa yanafanya kazi, ila kinachokuzuia kuona ni bendeji tu.

**Lini nitaweza kurudi kwenye kazi zangu za kawaida baada ya upasuaji mdogo?**

Baada ya kutoa bendeji, utaamua mwenyewe utakapopenda kuanza kufanya kazi zako za kawaida. Kwa kawaida watu wengi wanapumzika kwa siku moja au mbili. Bendeji ikishatolewa unaweza kabisa kuendelea na shughuli zako za kawaida kwa mfano kupika na kuchota maji. Utaweza kwenda shambani baada ya siku chache.

**Ni kwa muda gani nitahitaji msaada kutoka kwa muuguzi baada ya upasuaji mdogo?**

Siku ambayo utahitaji msaada kutoka kwa muuguzi ni siku ile uliyofanyiwa upasuaji TU. Utahitaji msaada wa kupelekwa nyumbani na kupika kwa sababu bendeji ulizofungwa zitakuwa zinakuzuia kuona. Mara bendeji zitakapoondolewa utakuwa una uwezo wa kufanya shughuli zako mwenyewe na hutahitaji msaada tena kutoka kwa muuguzi.

**Nitafanyaje kama ninaishi peke yangu?**

Kama unaishi peke yako ni muhimu kumtafuta mtu yeyote au ndugu yako alie karibu akusaidie kwa siku ile utakayopata upasuaji. Kesho yake baada ya kutolewa bendeji hutahitaji tena mtu wa kukusaidia. Kumbuka kwamba ukikataa kurekebishwa kope, baada ya muda mfupi tu unaweza kuwa na upofu, na utapata shida kubwa zaidi kwa muda mrefu kuliko siku ambayo utazuiwa kuona kwa ajili ya bendeji.

**Ni lini nahitaji kumuona daktari baada ya upasuaji mdogo?**

Baada ya wiki mbili inabidi urudi kwenye kituo cha afya ulichoelekezwa ili uondolewe nyuzi. Hata kama wiki mbili hazijafika na nyuzi bado unazo, haziathiri chochote kwenye jicho lako na utaweza kufanya kazi zako kama kawaida. Endapo utaona una tatizo lolote baada ya upasuaji, utaweza kwenda kwenye zahanati yako muda wowote.

**Kwa nini kufanyiwa upasuaji ni muhimu kuliko kupaka dawa au kung’oa kope zilizojikunja ndani ya jicho?**

Kupaka dawa kunaweza kusaidia kuondoa maumivu lakini haitasaidia kuondoa tatizo la kope kujikunja na kuingia ndani ya jicho na wala haitazuia jicho kupata upofu. Kung’oa kope hakumalizi tatizo kwa sababu kope zinapoota tena zinaweza kuchoma jicho lako tena. Tiba ya kudumu ni upasuaji ambao utarekebisha kope, utaondoa maumivu na pia utazuia upofu wa macho yako.

**Huu upasuaji mdogo utamuwezesha mtu kipofu kuona tena?**

HAPANA. Upasuaji huu hauondoi upofu ila unazuia kupata upofu na kurudisha kuona kwa wale ambao si vipofu kabisa. Wakati mwingine watu ambao ni vipofu hupenda kufanyiwa upasuaji ili kuondoa maumivu ya jicho na machozi ambayo hutoka mara kwa mara.

**Upasuaji utaumiza?**

Mwanzoni kabisa kabla ya upasuaji utapata sindano ambayo inauma kidogo, baada ya hapo hutasikia maumivu yoyote wakati upasuaji ukiendelea. Kifuniko cha jicho kinaweza kuuma au kuvimba kidogo kwa siku chache baada ya upasuaji. Ukipenda, unaweza kunywa panadol kuzuia tatizo hilo.

**Upasuaji una gharama zozote kwa mgonjwa?**

HAPANA. Upasuaji na dawa zote utakazopewa baada ya upasuaji hutolewa bure.

**Upasuaji huu utarekebisha matatizo yote ya macho?**

Kuna matatizo mengi sana ya macho. Upasuaji huu unatatua tatizo la kope kujikunja kuelekea ndani ya jicho. Matatizo mengine kama presha ya jicho au mtoto wa jicho hatuwezi kuyatatua kijijini kwa sababu yanahitaji kuchunguzwa kwa kutumia mashine maalum, umeme, nk. Kama mgonjwa ana matatizo hayo, inabidi aende hospitali kama Ndanda au Ligula. Kama wewe una vikope na pia una matatizo mengine katika macho yako, sisi tutaweza kutibu vikope TU hapa kijijini.
